# Supplementary material for: Facilitating NMR Resonance Assignment with Metabolic Tampering
Source: bioRxiv. 2026 May 1:2026.04.29.721603. Preprint. [Version 1] doi: 10.64898/2026.04.29.721603 (PMC13142442; doi:10.64898/2026.04.29.721603)
Supplement: Supplement 1 [file media-1.pdf]

Supporting Information

Facilitating NMR Resonance Assignment with Metabolic Tampering

Danica S. Cui,[1] Evan O. Anderson,[2] Erik Zavala,[3,5] George P. Lisi,[1,4] and J. Patrick Loria[1,3]*

[1]Department of Chemistry, Yale University, New Haven, CT 06520
[2] Department of Cellular and Molecular Physiology, Yale University, New Haven, CT 06520
[3]Department of Molecular Biophysics & Biochemistry, Yale University, New Haven, CT 06520

[4]Current Address: Department of Molecular Biology, Cell Biology & Biochemistry, Brown University, Providence, RI 02903
[5]Current Address: Oxford Instruments, 300 Baker Avenue, Suite 150, Concord, MA 01742

*Corresponding author: patrick.loria@yale.edu, 203-436-2518

Table of Contents

Figure S1: O.D. growth curve of *E.coli* in different combinations of LB and M9 media

Figure S2: Summary of normalized peak intensity changes for amino acids in IGPS

Figure S3: Control experiment for the effect of ^{1}H-induced relaxation on peak intensities for IGPS

Figure S4: Control experiment for the effect of ^{1}H-induced relaxation on peak intensities for PTP1B

Figure S5: T_1 measurements for 10% (H_2O, ^{14}N) and 10% LB IGPS

Figure S6: T_2 estimations from line width analysis

Figure S7: Extrapolation of peak intensity changes from 1% IGPS data

Figure S8: Effects of LB doping on $^{13}CH_3$ HMQC NMR spectra of Ile, Leu, and Val methyl groups in IGPS

Figure S9: Empirical distribution function of signal intensity ratios in IGPS and PTP1B

Figure S10: Boxplots of $Z(I_{10}/I_0)$ and $Z(I_{10}/I_1)$ values for PTP1B and IGPS

Figure S11. PHPT1 test data amino acid hits based on $Z(I_{10}/I_1)$ values

Figure S12: Confusion matrices of additional random forest models for amino acid classification

Figure S13: Comparison of Z-scores for residues with overlapping ^{13}Cβ chemical shifts

Figure S14: The signal to noise of NMR resonance peaks observed in 10% LB ^{15}N HSQC

Figure S15: Schematic of the user–end process on using random forest MeTA prediction tool

Table S1: $Z(I_{10}/I_1)$ values of PHPT1 test data (n=34) . Protein was expressed at 25ºC for 16 hr.

SI Methods

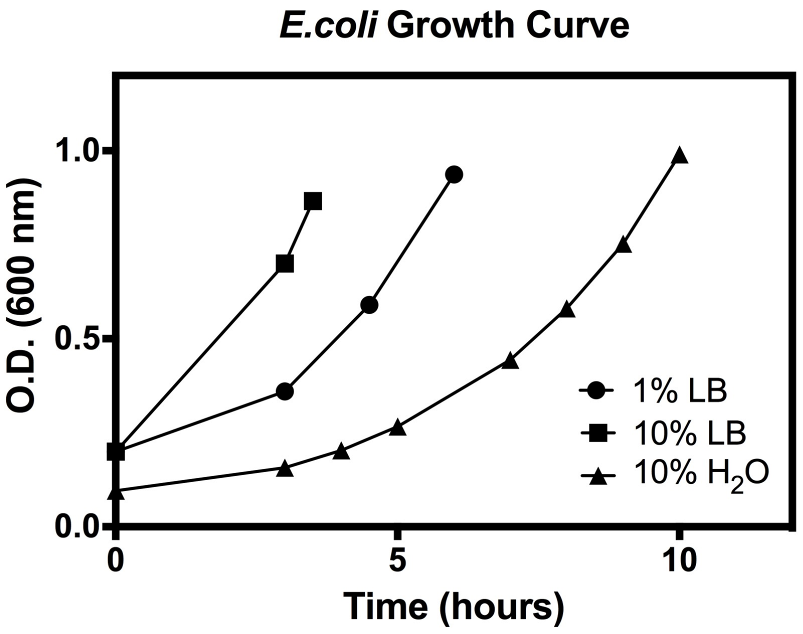

Figure S1. O.D. growth curve of *E.coli* BL21-CodonPlus (DE3)-RIL cells in D_2O M9 minimal media with 1% LB, 10% LB, and 10% H_2O.

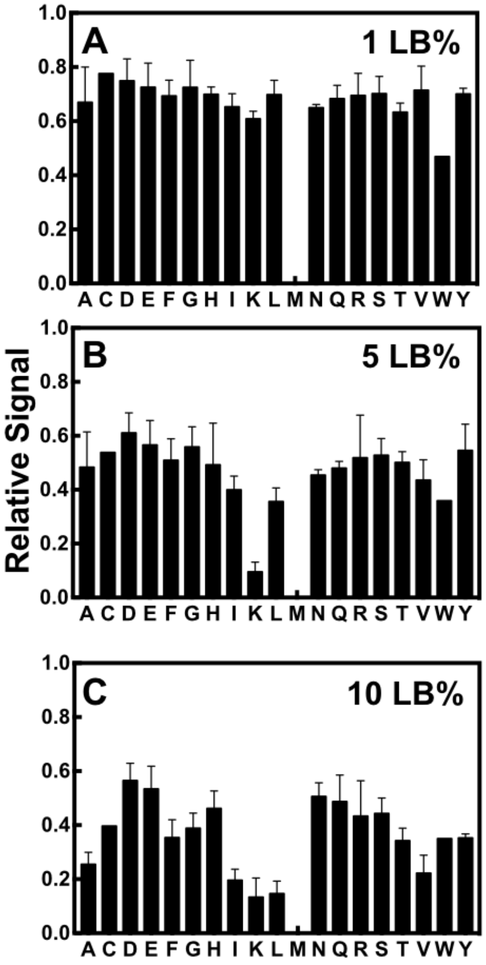

Figure S2. Summary of normalized peak intensity changes for amino acids in the HisF subunit of IGPS determined from ^{1}H-^{15}N TROSY experiments in which 1% **(A)**, 5% **(B)**, or 10% **(C)** LB medium was incorporated into the bacterial culture.

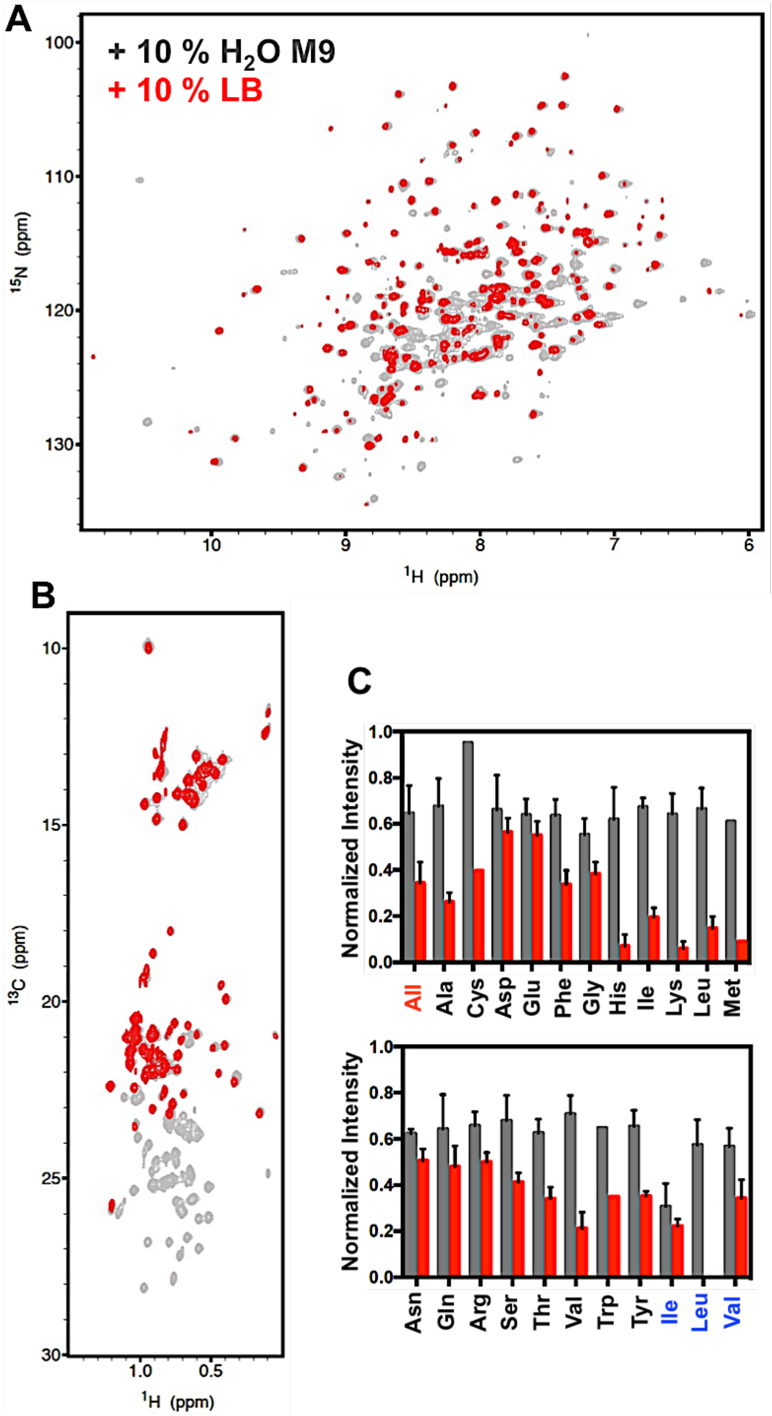

Figure S3. Control experiment (expressed for 8hr at 37ºC) assessing the contribution of ^{1}H-induced dipolar relaxation to the attenuation of NMR signal intensities. **(A)** ^{1}H-^{15}N TROSY spectral overlays comparing identical samples of IGPS where 10% by volume of 100% ($^{1}H_2O$, ^{14}N) -M9 minimal mediums (gray) or 10% LB medium (red) was incorporated into the bacterial protein expression culture. **(B)** ^{1}H-$^{13}CH_3$-ILV HMQC spectral overlays comparing the same experimental conditions described in **(A)**. **(C)** Summary of residual peak intensities for every non-proline amino acid in the presence of 10% H_2O M9 (gray) and 10% LB (red). Values are also reported for the

entire data set (red text) and for Ile, Leu, and Val residues in ^1H-$^{13}CH_3$ HMQC experiments shown in **(B)** (blue text)

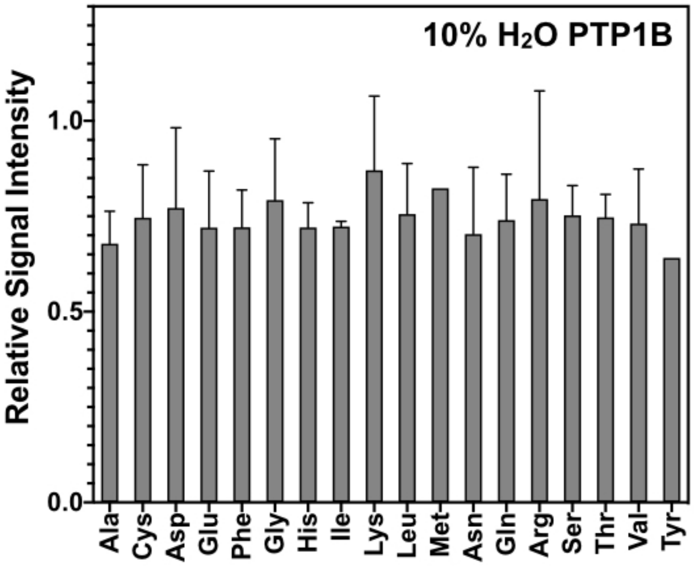

Figure S4 – PTP1B control experiment (expressed for 8hr at 37ºC) assessing the contribution of ^1H-induced dipolar relaxation to the attenuation of NMR signal intensities. Summary of relative peak intensity in the presence of 90%D_2O/10% H_2O M9 compared to ^{15}N labeled PTP1B grown in 100% D_2O M9.

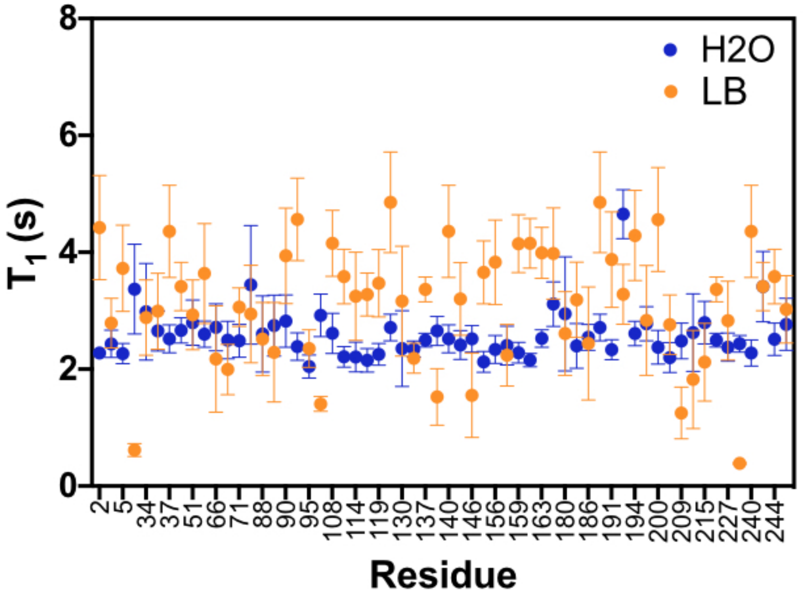

Figure S5 – T_1 values measured for ^{15}N IGPS sample grown in 10% (H_2O, ^{14}N) and 10% LB perdeuterated M9 media. The average T_1 values for 10% (H_2O, ^{14}N) sample is 2.6 s ± 0.4, and for 10% LB it is 3.1 s ± 1.

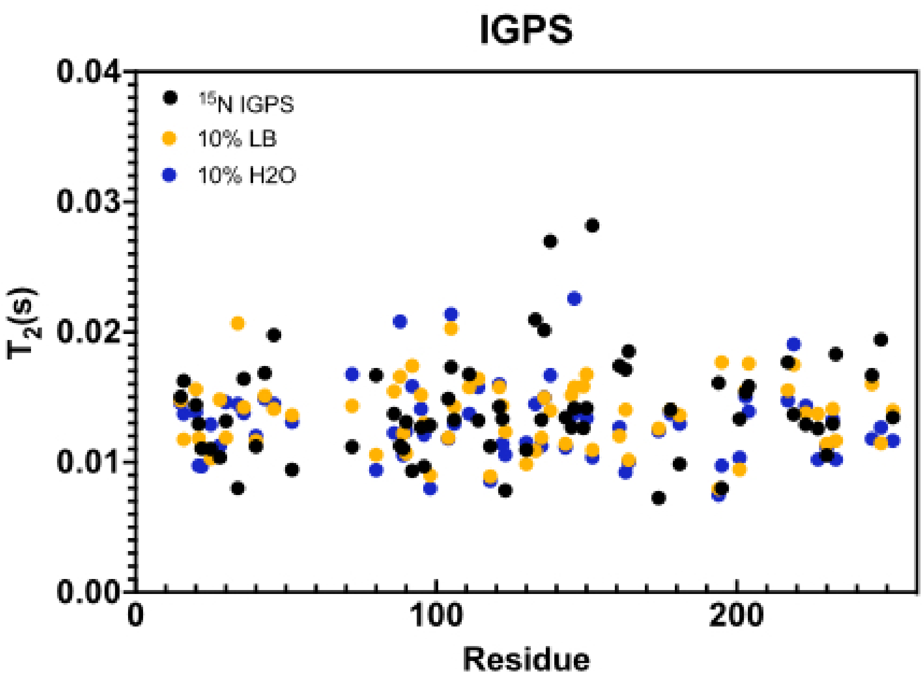

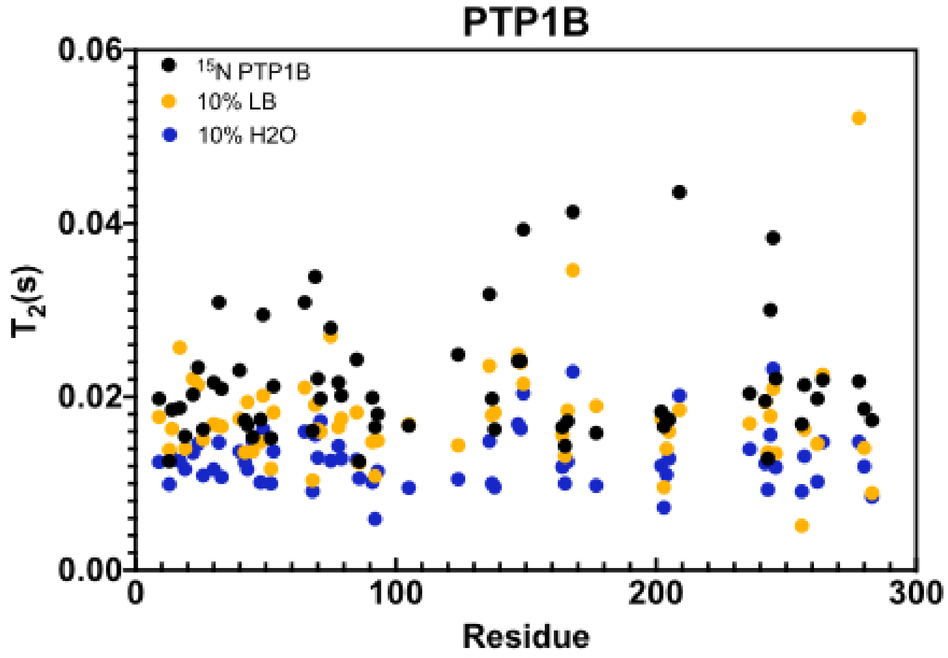

Figure S6 – T_2 values estimated from NMR resonance peak line width measurements for ^{15}N IGPS and PTP1B samples grown in 100% D_2O M9 media, with 10% H_2O, and 10% LB addition. Line widths at half height ($\nu_{1/2}$) are determined from integration of NMR resonance peaks with a Lorentzian fit in NMRFAM-Sparky. T_2 values are calculated from the following equation: $\nu_{1/2}=1/(\pi \times T_2)$. The average T_2 values for fully perdeuterated growth of IGPS and PTP1B are 0.014 ± 0.04 and 0.021 ± 0.007 respectively. For 10% H_2O sample, the IGPS and PTP1B T_2 values were determined to be 0.014 s ± 0.003 and 0.013 ± 0.003, and for 10% LB it is 0.013 s ± 0.003 and 0.018 s ± 0.006 respectively.

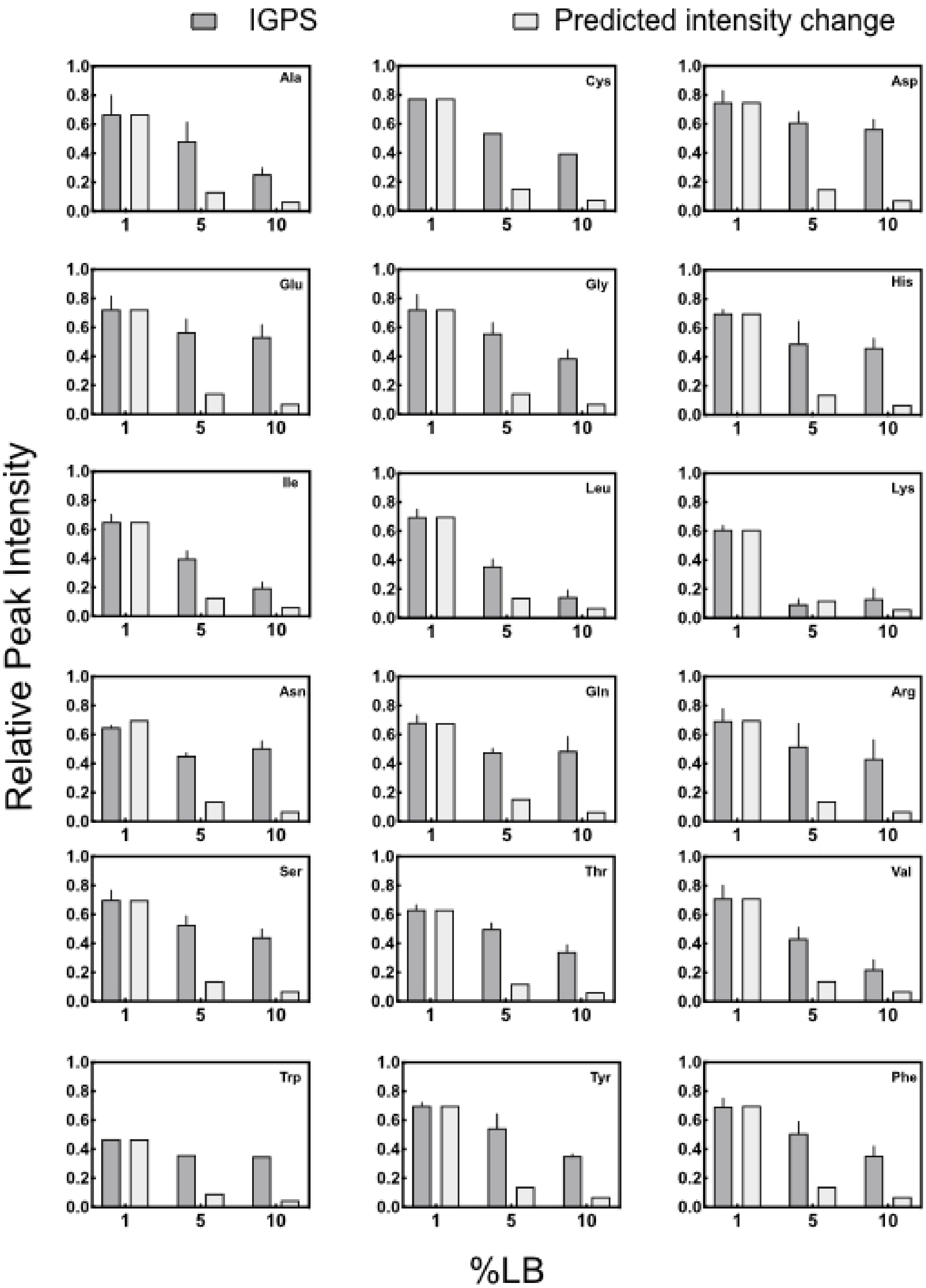

Figure S7. Predicted relative intensity changes (light grey) for 5% and 10% LB experiments extrapolated from 1% IGPS data compared to real values obtained from the 5% and 10% LB NMR experiments (dark grey). This analysis reveals that the NMR signal attenuation for Lys is LB concentration dependent. Glu and Asp residues have the highest signal retention. This is because the Glu biosynthetic pathway is responsible for the assimilation of inorganic nitrogen into other amino acid biosynthetic pathways. Additionally, both Glu and Asp are substrates for transamination reactions for all other amino acid biosynthesis pathways (shown in Fig. 1).

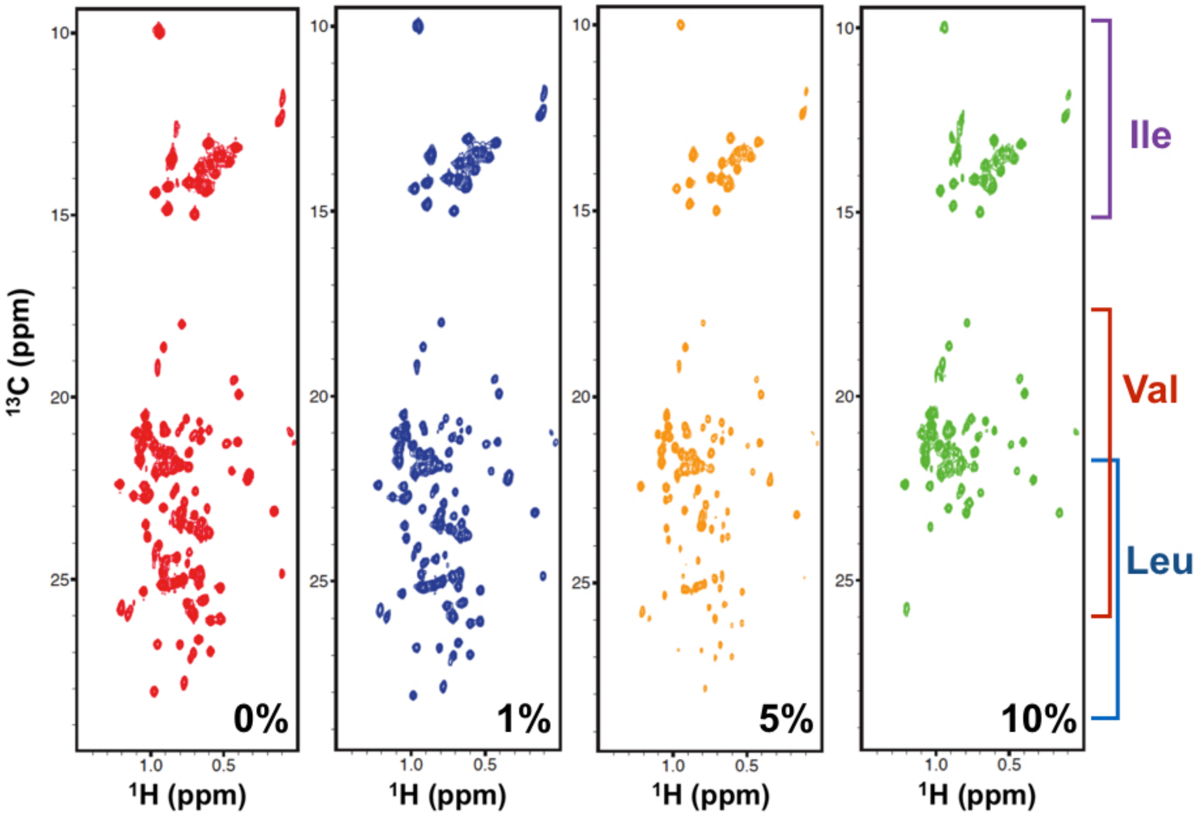

Figure S8. Effects of LB doping on ILV spectra. $^{1}H^{13}CH_3$ HMQC NMR spectra of Ile, Leu, and Val methyl groups in IGPS depicting changes in peak intensities in the presence of 1% (blue), 5% (orange), and 10% by volume, LB (green) in the growth medium, relative to that of a reference spectrum (red) without added LB. Leu resonances are strongly affected by LB and disappear gradually from the spectrum and altogether at 10% LB.

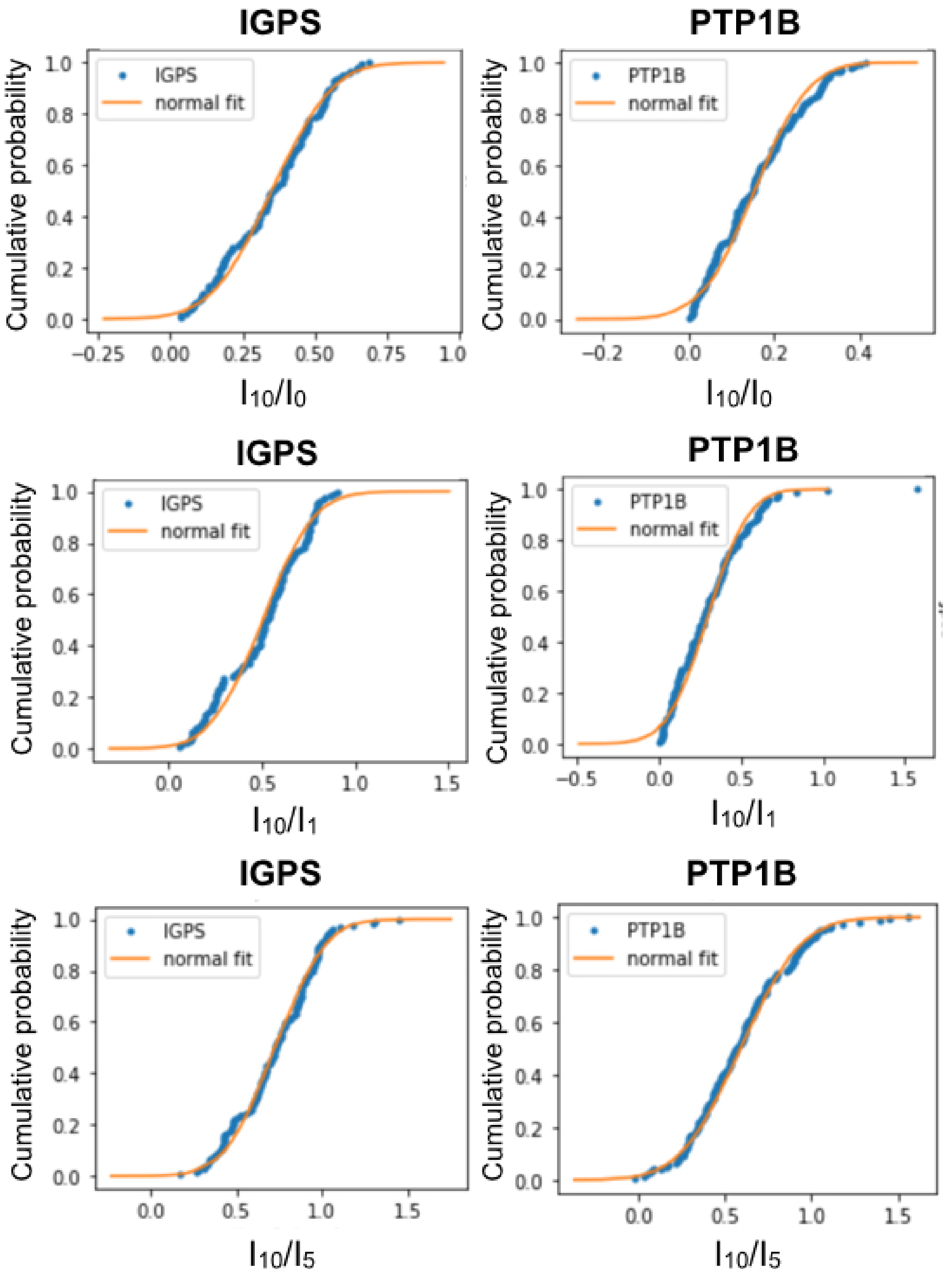

Figure S9. The empirical distribution function for signal intensity ratios of 10% LB over 0%, 1% and 5% LB is shown for IGPS and PTP1B. These signal ratios are shown to have a normal distribution.

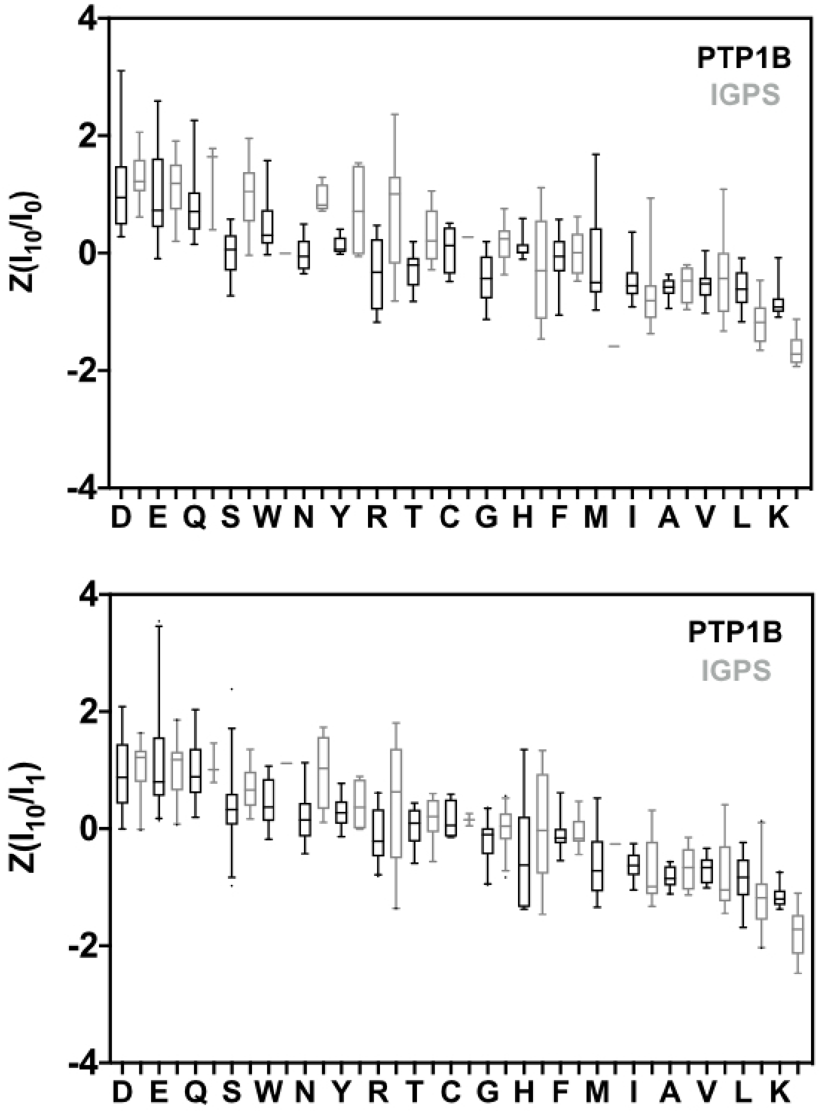

Figure S10. Boxplots of $Z(I_{10}/I_0)$ and $Z(I_{10}/I_1)$ values in the order of high to low median values shown for the 8hr and 20hr growths of IGPS and PTP1B. The box represents the interquartile range (25–75 percentile), the median is shown with a line, and the whiskers represent 1-99 percentile range.

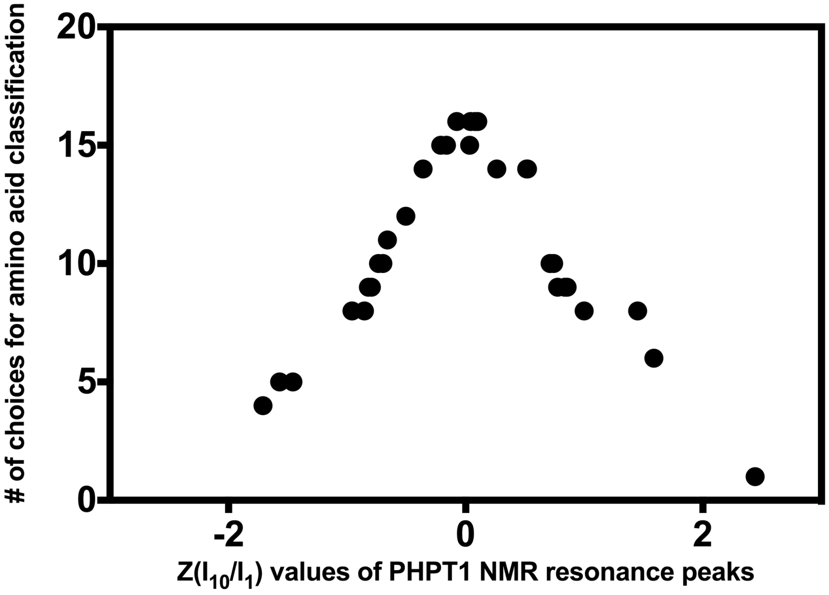

Figure S11. The distribution of the number of potential amino acid assignments for residues with $Z(I_{10}/I_1)$ values that are within range of the PHPT1 test data set plotted against the $Z(I_{10}/I_1)$ values of the PHPT1 test data set. The data clearly shows the ambiguity of amino acid type assignment for residues with $-0.7 < Z(I_{10}/I_1) < 0.7$.

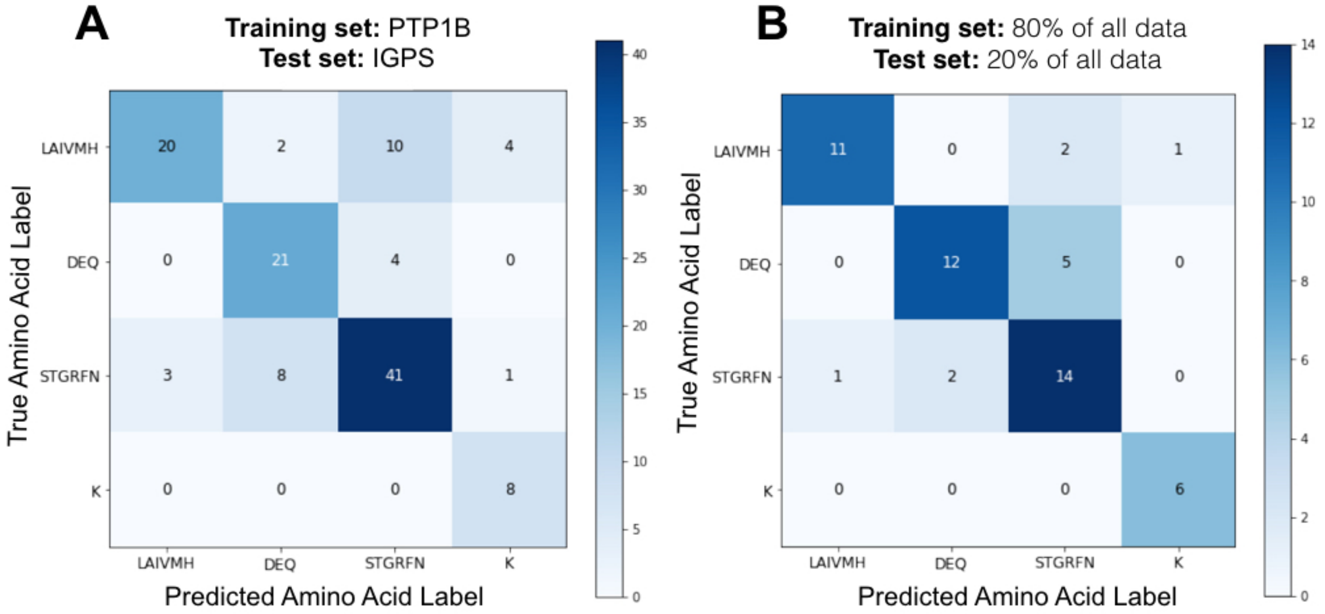

Figure S12. Confusion matrices summarizing the prediction power for **A)** a model trained on PTP1B data (n=147) and tested on IGPS data (n=122), where the resulting accuracy was 74%, and **B)** a model trained on combined data in a 80% /20% split of the training and test data. This model had an accuracy of 80%.

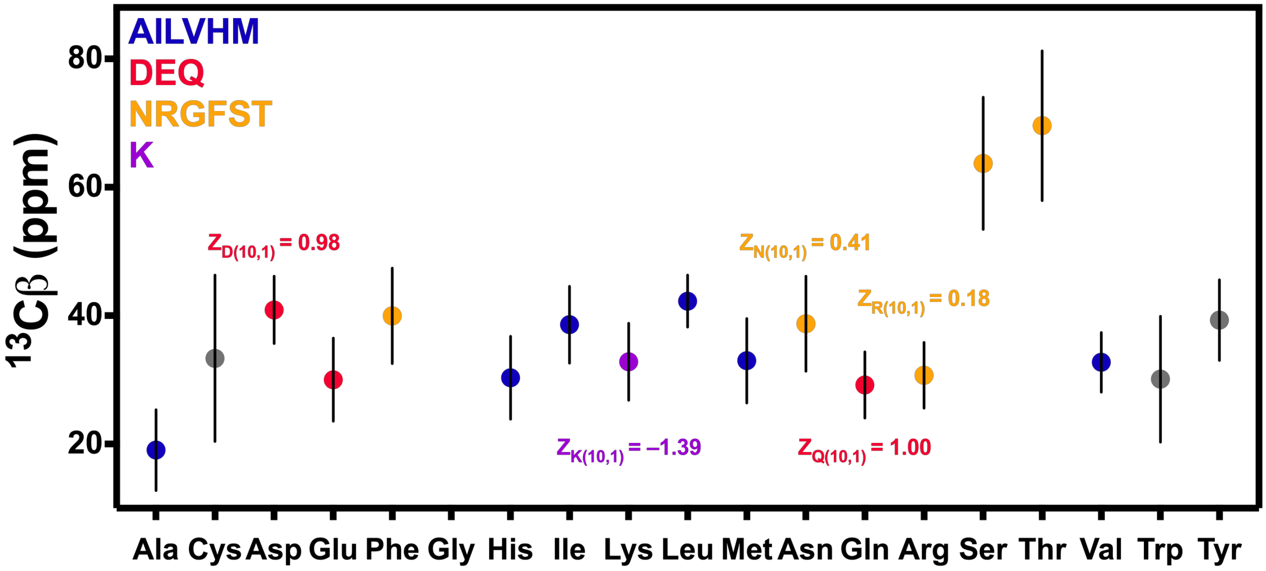

Figure S13. The average and standard deviation of ^{13}Cβ chemical shifts obtained from the Biological Magnetic Resonance Bank (BMRB). LB doping experiments enable the separation of amino acid types with degenerate Cβ chemical shifts. Z(I_{10}/I_1) values for select residues are shown above that amino acid type.

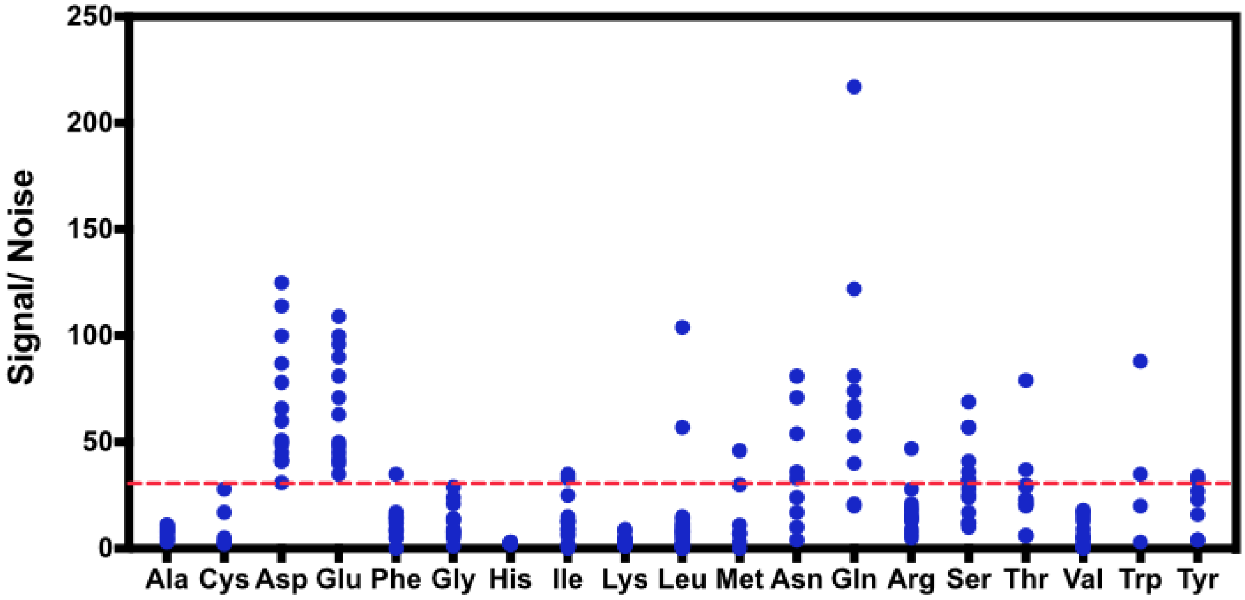

Figure S14. The signal to noise of NMR resonance peaks observed in 10% LB ^{15}N HSQC spectrum of 336 µM PTP1B. A total of 67 residues are observed with S/N > 30 (indicated with a red line).

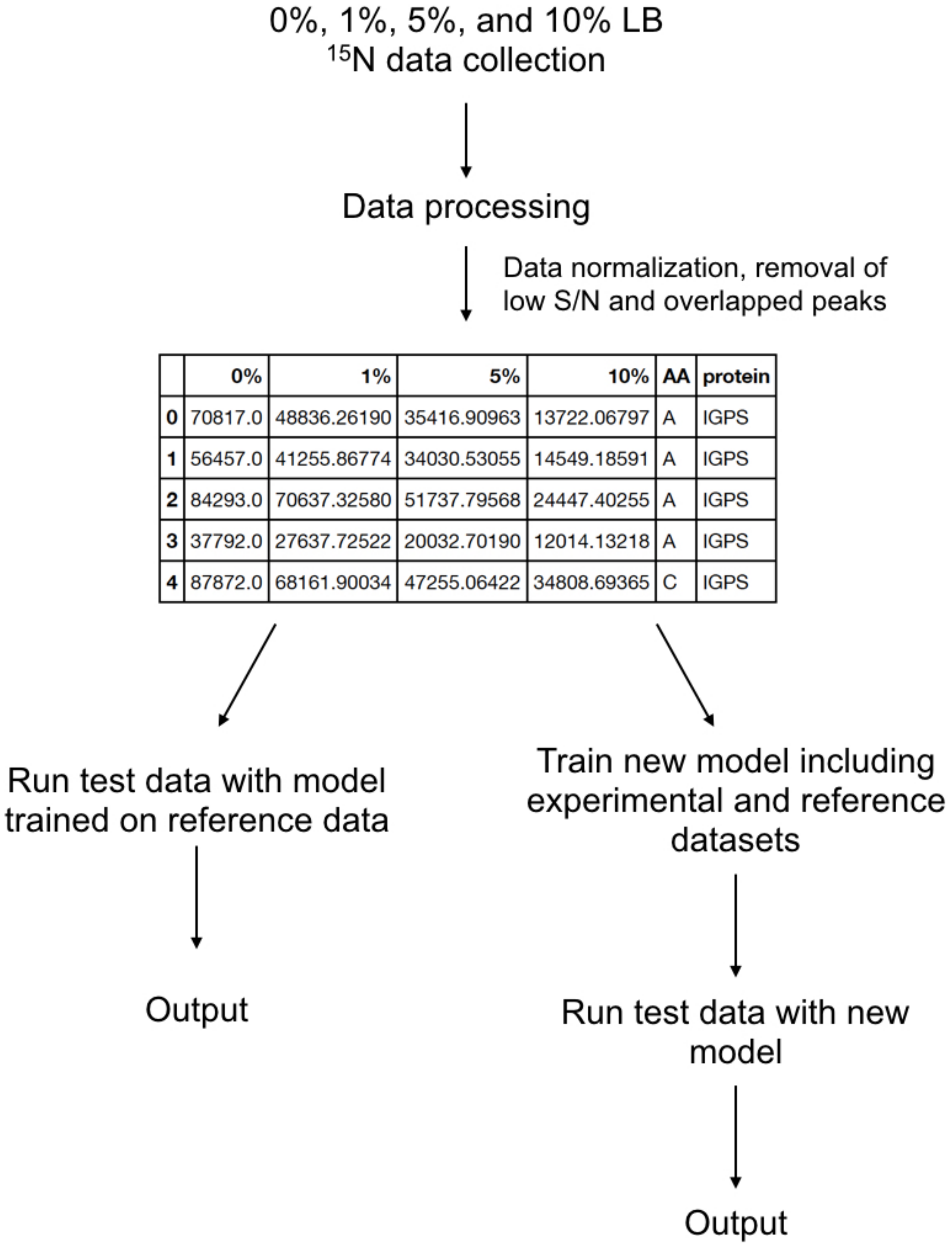

	0%	1%	5%	10%	AA	protein
0	70817.0	48836.26190	35416.90963	13722.06797	A	IGPS
1	56457.0	41255.86774	34030.53055	14549.18591	A	IGPS
2	84293.0	70637.32580	51737.79568	24447.40255	A	IGPS
3	37792.0	27637.72522	20032.70190	12014.13218	A	IGPS
4	87872.0	68161.90034	47255.06422	34808.69365	C	IGPS

Figure S15. Suggested workflow for usage of MeTA prediction tool. It is recommended that all NMR experiments be conducted with the same parameters. The model optimization documentation is available at github (https://github.com/evan-anderson/MeTA) .

Table S1. $Z(I_{10}/I_1)$ values of PHPT1 test data (n=34) . Protein was expressed at 25ºC for 16 hr.

	$Z(I_{10}/I_1)$		$Z(I_{10}/I_1)$
**A50**	-0.795	**R45**	0.521
**A54**	-0.959	**R78**	0.515
**C73**	-0.211	**S61**	0.714
**D58**	2.298	**S80**	0.837
**E51**	1.450	**S94**	0.743
**E72**	1.587	**T106**	0.711
**G62**	0.078	**V23**	-0.6o61
**G75**	0.262	**V27**	-0.853
**G77**	0.043	**V44**	-0.820
**G92**	0.102	**V90**	-0.958
**G98**	-0.076	**Y47**	0.999
**H102**	-0.735	**K110**	-1.569
**I43**	-0.359	**V118**	-0.698
**K41**	-1.709	**T119**	0.775
**K48**	-1.458	**N122**	0.856
**L6**	-0.504	**G124**	-0.162
**R26**	0.033	**L74**	-0.359

SI Methods:

IGPS $^{13}CH_3$ ILV labeling experiment

Separate samples of IGPS were produced in which isotopic labeling of isoleucine, leucine, and valine (ILV) methyl groups was achieved by adding 60 mg/L of alpha-ketobutyric acid [methyl-^{13}C;3,3-D2] and 100 mg/L of alpha-ketoisovaleric acid [3-methyl-^{13}C;3,4,4,4-D2] (Cambridge Isotope Labs) 30 minutes prior to induction.(Tugarinov et al. 2006) Cells were allowed to reach an OD_{600} of 0.8 – 1.0 before induction with 1 mM IPTG. Cells were expressed under two post-induction conditions: 8 hrs at 37 °C, and 20 hrs at 20ºC. The cells (containing isotopically labeled HisF and perdeuterated HisH) were harvested by centrifugation and resuspended in a buffer of 10 mM Tris, 10 mM CAPS, 300 mM NaCl and 1 mM β-mercaptoethanol at pH 7.5 and then co-lysed by ultrasonication. The cell lysate was clarified by centrifugation and the IGPS complex was purified by Ni-NTA affinity chromatography utilizing the C-terminal histidine tag on HisH. IGPS samples for NMR study were concentrated to 0.38 – 0.41 mM in a buffer containing 10 mM HEPES, 10 mM KCl, 0.5 mM EDTA, and 1 mM DTT at pH 7.3.

Experiments probing the side chain methyl groups of Ile, Leu, and Val ($^{13}CH_3$-ILV) were based on the multiple-quantum (MQ) pulse sequence.(Mueller 1979) In these experiments, transmitter offsets alternated between the water resonance and the center of the methyl region (0.75 ppm), while the ^{13}C channel offset was set to 19.5 ppm. All $^{1}H^{13}CH_3$-ILV spectra were collected with 32 transients, 144 t_1 increments, 3778 data points, and spectral widths of 8500 Hz (direct) and 3500 Hz (indirect). NMR spectra were processed with NMRPipe (Delaglio et al. 1995) and analyzed in PINE-SPARKY (Lee et al. 2009). NMR resonance signal intensities in 0%LB PTP1B were multiplied by two to account for the collected transient differences between experiments.

References:

Delaglio, F., S. Grzesiek, G. W. Vuister, G. Zhu, J. Pfeifer and A. Bax (1995) NMRPipe: A Multidimensional Spectral Processing System Based on UNIX Pipes. J. Biomol. NMR 6(3): 277

Lee, W., W. M. Westler, A. Bahrami, H. R. Eghbalnia and J. L. Markley (2009) PINE-SPARKY: graphical interface for evaluating automated probabilistic peak assignments in protein NMR spectroscopy. Bioinformatics 25(16): 2085.10.1093/bioinformatics/btp345

Mueller, L. (1979) Sensitivity Enhanced Detection of Weak Nuclei using Heteronuclear Multiple Quantum Conherence. J. Am. Chem. Soc. 101: 4481

Tugarinov, V., V. Kanelis and L. E. Kay (2006) Isotope Labeling Strategies for the Study of High-Molecular-Weight Proteins by Solution NMR Spectroscopy. Nat. Protoc. 1(2): 749.10.1038/nprot.2006.101
